# Medical Education

**Published:** 1860-08

**Authors:** 


**Medical Education.**

The Editor of the American Medical Gazette very properly remarks, in reference to the late action of the Convention of Medical Teachers and American Medical Association on the subject of Medical Education: "After all that has been professed and written on this subject by leading men in the profession—and this during so many years, and in most of the periodicals of the country—it would seem apparent, from recent developments, that no improvement or reform has ever been seriously entertained, other than in mere sentimental flourishes." This does not, however, develope the matter fully. In addition to a demonstration of the fact that the leading men in this cry of reform were not sincere in desiring any *real* or radical change or improvement in Medical Education, we have had exhibited the true animus of the whole thing—"the *kernel* of the matter," as one of the leaders unwittingly admitted, when at one time his long cherished purpose seemed about to fail—the determination to kill off Summer Schools by the adoption of a senseless requisition of twelve month's interval between courses of Lectures, even though the student may have been engaged in study three, four, or any number of years. That this was the true object of these pseudo reformers, and that they were not actuated by any real desire for the elevation of the standard of Medical Education, has been clearly exhibited by the utter indifference (amounting almost to contempt) with which they treated the able report of Dr. Reese, the Chairman of the Standing Committee on Medical Education, in which was contained suggestions for some real and important changes in our present defective system, and still more strikingly manifesting their insincerity by pressing with the greatest pertinacity what we have for years charged upon them, as being the real motive at the bottom of this new-born zeal in the cause of Medical Education.

